# Kinetic Study and Reaction Mechanism of the Gas-Phase Thermolysis Reaction of Methyl Derivatives of 1,2,4,5-Tetroxane

**DOI:** 10.3390/molecules29143274

**Published:** 2024-07-11

**Authors:** Alexander G. Bordón, Mariela I. Profeta, Jorge M. Romero, María J. Jorge, Lilian C. Jorge, Nelly L. Jorge, C. Ignacio Sainz-Díaz, Juliana Cuéllar-Zuquin, Daniel Roca-Sanjuán, César Viseras Iborra, André Grand, Alfonso Hernández-Laguna

**Affiliations:** 1 Laboratorio de Investigaciones en Tecnología Ambiental, Área de Química Física Facultad de Ciencias Exactas y Naturales y Agrimensura, Universidad Nacional del Nordeste, Av. Libertad 5460, Corrientes 3400, Argentina; germanbordon_7@hotmail.com (A.G.B.); marielaprofeta@hotmail.com (M.I.P.); ing.jorgemromero@gmail.com (J.M.R.); maria.jorge@comunidad.unne.edu.ar (M.J.J.); lilian.jorge@vet.unne.edu.ar (L.C.J.); lidianj@exa.unne.edu.ar (N.L.J.); 2 Instituto Andaluz de Ciencias de la Tierra, Consejo Superior de Investigaciones Científicas (CSIC), Av. Las Palmeras 4, 18100 Armilla, Granada, Spain; cviseras@ugr.es; 3 Instituto de Ciencia Molecular, Universitat de València, Apartado 22085, 46071 Valencia, Spain; maria.juliana.cuellar@uv.es (J.C.-Z.); daniel.roca@uv.es (D.R.-S.); 4 Department of Pharmacy and Pharmaceutical Technology, Faculty of Pharmacy, University of Granada, Campus de Cartuja s/n, 18071 Granada, Spain; 5 Unité de Formation et de Recherche, Université Grenoble Alpes, Commissariat á l’Énergy Atomique (CEA), Centre National de la Recherche Scientifique (CNRS), Institute for Nanoscience and Cryogenics-Systèmes Moléculaires et Nanomatériaux por l’Énergieset la Santé (INAC-SyMMES), 38000 Grenoble, France; andre.grand8@wanadoo.fr

**Keywords:** antimalaric drugs, quantum-mechanical calculations, methyltetroxanes, spin orbit coupling, thermolysis mechanism

## Abstract

Tetroxane derivatives are interesting drugs for antileishmaniasis and antimalaric treatments. The gas-phase thermal decomposition of 3,6,-dimethyl-1,2,4,5-tetroxane (DMT) and 3,3,6,6,-tetramethyl-1,2,4,5-tetroxane (acetone diperoxide (ACDP)) was studied at 493–543 K by direct gas chromatography by means of a flow reactor. The reaction is produced in the injector chamber at different temperatures. The resulting kinetics Arrhenius equations were calculated for both tetroxanes. Including the parent compound of the series 1,2,4,5-tetroxane (formaldehyde diperoxide (FDP)), the activation energy and frequency factors decrease linearly with the number of methyl groups. The reaction mechanisms of ACDP and 3,6,6-trimethyl-1,2,4,5-tetroxane (TMT) decomposition have been studied by means of the DFT method with the BHANDHLYP functional. Our calculations confirm that the concerted mechanism should be discarded and that only the stepwise mechanism occurs. The critical points of the singlet and triplet state potential energy surfaces (S- and T-PES) of the thermolysis reaction of both compounds have been determined. The calculated activation energies of the different steps vary linearly with the number of methyl groups of the methyl-tetroxanes series. The mechanism for the S-PES leads to a diradical O···O open structure, which leads to a C···O dissociation in the second step and the production of the first acetaldehyde/acetone molecule. This last one yields a second C···O dissociation, producing O_2_ and another acetone/acetaldehyde molecule. The O_2_ molecule is in the singlet state. A quasi-parallel mechanism for the T-PES from the open diradical to products is also found. Most of the critical points of both PES are linear with the number of methyl groups. Reaction in the triplet state is much more exothermic than the singlet state mechanism. Transitions from the singlet ground state, S_0_ and low-lying singlet states S_1–3_, to the low-lying triplet excited states, T_1–4_, (chemical excitation) in the family of methyl tetroxanes are also studied at the CASSCF/CASPT2 level. Two possible mechanisms are possible here: (i) from S_0_ to T_3_ by strong spin orbit coupling (SOC) and subsequent fast internal conversion to the excited T_1_ state and (ii) from S_0_ to S_2_ from internal conversion and subsequent S_2_ to T_1_ by SOC. From these experimental and theoretical results, the additivity effect of the methyl groups in the thermolysis reaction of the methyl tetroxane derivatives is clearly highlighted. This information will have a great impact for controlling these processes in the laboratory and chemical industries.

## 1. Introduction

Organic peroxides are a series of compounds with many different applications, due to their therapeutic properties and explosive character. Some of them are used to fight malaria in subtropical regions, and as antibiotic; some others therapeutical properties are the intervention in biological metabolites, cancer illness, cellular aging, and herbicide use [1,2,3,4,5].

Furthermore, commercial vinyl polymers—such as polystyrene, polyethylene, and polyvinyl chloride—are produced industrially through free radical polymerization, where organic peroxides are commonly used as free radical initiators. The thermal decomposition of organic diperoxides through the homolytic cleavage of the peroxide bond generates a biradical intermediate, which would initiate the polymerization of vinyl monomers. This biradical species would be incorporated into the polymer structure, giving polymeric species that contain peroxide groups in their structures, which are then decomposed in the next processes. From the structure of the organic peroxide, a biradical is generated, which influences on the properties of the polymer. Therefore, it is important to clarify the decomposition reaction mechanism of the di- and triperoxide organic compounds, which can be considered multifunctional initiators due to the presence of more than one peroxide group [1]. However, the knowledge of the behavior and reaction mechanism of these compounds is limited. Hence, a further understanding of these processes would allow the control of many industrial processes with high impact in economic costs, and environmental effects.

The tetroxane-derivative antimalaric drugs are compounds with large molecular weight, low toxicity and no genetic toxicity. It is well known that the diperoxide compounds are more suitable as drugs than the equivalent ozonides and trioxane compounds [2]. Therefore, the diperoxide compounds are considered as new anti-malaric drugs and can be a good choice in front of artemisinin derivative drugs [2,3,4]. A deeper knowledge of the reactivity of these compounds close to receptor environment would help in the design of optimal antimalaric drugs.

Diperoxide compounds are also found with an important herbicide activity [6]. Many tetroxanes have got phyto-toxicity, inhibiting the growing of roots, especially cucumber and sorghum [6]. This activity is statistically comparable to the glyphosate and imazapyr [6]. One of the compounds of this family (3,3,6,6-tetramethyl-1,2,4,5-tetroxane—in short, either acetone diperoxide (ACDP) or tetramethyl-tetroxane), which is of interest in the present work, has shown especially high phytotoxicity. 

In previous papers, the experimental kinetic and theoretical mechanism of the thermolysis reaction at gas phase of the parent compound of the series (1,2,4,5-tetroxane (formaldehyde diperoxide, FDP)) was accomplished [7]. Additionally, the theoretical mechanism of the thermolysis reaction of 3-methyl-1,2,4,5-tetroxane (MFDP) [8] and 3,6-dimethyl-1,2,4,5-tetroxanes (DMT) [9] was also determined. Previously, a concerted mechanism of the thermolysis reaction was proposed [10], but from quantum mechanics calculations, a stepwise mechanism was also determined. In these previous works [7,8,9], a common stepwise mechanism for the thermolysis reaction was found with lower activation energy than the previously proposed concerted mechanism. In the stepwise mechanism, the first step brings the reactant to a diradical open (**o**) intermediate in its singlet state (S_0_), and, afterwards two step-wise mechanisms follows: (i) a decomposition step by step from the **o** structure to two aldehydes molecules plus an oxygen molecule in its excited singlet state (S_1_); and (ii), a transition from the S-**o** diradical structure to an **o** structure in its triplet state (T_1_), and a subsequent triplet stepwise mechanism, producing two molecules of aldehydes plus one oxygen molecule in its ground triplet state (T_1_). This singlet-to-triplet-state non-adiabatic transition is deeply studied in this work. In general, the rate-limiting step was the second step. The thermal decomposition mechanism in dioxetane was also found to proceed in a stepwise manner and with a non-negligible nonadiabatic transition from the singlet to triplet state [11]. 

The methyl group substitution effect was analyzed previously [9], showing that the activation energies (*Ea*), reaction energies (*Er*), and reaction coordinate lengths of the different steps of the stepwise mechanism of the reaction behave linearly with the number of methyl groups. 

Because of the peroxide bonds and the cyclic structure, these compounds become highly explosive. In general, they are compounds of an unstable and explosive nature due to the peroxide groups, which are intrinsically unstable and highly reactive [5], so the synthesis and manipulation of these materials require strict care to avoid accidents. The multiple incidents recorded in recent decades involving this type of species have raised the need for a constant and exhaustive physicochemical study. Methyl derivatives are especially dangerous because of their high explosivity, and their synthesis and manipulation should be performed in very small amounts. Additionally, the products formed during the thermolysis reaction of the tetroxane derivatives augment three times the number of moles with respect to the reactants, contributing also to their high-explosive character. The successive methylation of the tetroxanes yields different reaction heats. These features plus the surplus incentive of knowing the kinetic and mechanism of thermolysis reaction of the highest and lowest methyl-substituted members of the series and its comparison with the parent compound increase the interest of this work. The knowledge of these complex non-adiabatic processes will facilitate the disposition of control techniques for further synthesis and manufacturing scale processes. This will have a great economic and environmental impact in the chemical industry. 

Herein, the following goals are pursued: (i) to carry out an experimental kinetic characterization of DMT and ACDP of the thermolysis reaction in the gas phase; (ii) to determine the theoretical thermolysis reaction mechanism of trimethyltetroxane (TMT) and ACDP; and (iii) to theoretically revisit tetroxane and its methyl derivatives in order to study the non-adiabatic transition occurring in the diradical open structure (chemiexcitation) by multiconfigurational quantum chemistry. The obtained data will be studied in order to know the methyl substitution effect in the gas phase thermolysis reaction of the methyl-tetroxanes.

## 2. Results and Discussion

### 2.1. Analytical Results

After the introduction of ACDP [10] into the injector chamber [12], only three compounds were detected: solvent (retention time = 1.3 min), acetone (2.84 min), and the remaining non-reacted ACDP (8.7 min). As a consequence of the very short residence time (40 s) of ACDP in the injector chamber, acetone was the only product detected, and no other products coming from possible secondary reactions were found.

The introduction of DMT into the injector chamber showed the detection of three compounds: acetaldehyde (1.4 min), the solvent (3.2 min), and the remaining non-reacted DMT (7.2 min). Acetaldehyde was the only product detected, a consequence of the very short residence time (40 s) of DMT in the injector chamber.

### 2.2. Kinetic Results

According to Arrhenius’s equation, the *Ea* was calculated from the slope of least-square fitting of *ln*[*ln*(*C*_0_/*C*)] as a function of 1/*T* and the intersect of this fitting yields the *ln*(*A.t*). Considering the retention time, which is constant in all measures, *A* can be obtained.

The rate constant of reaction, *k_exp_*, for DMT and ACDP at different temperatures (Table 1), gives the following Arrhenius equations:

*ln k_exp,DMT_ =* (25.66 ± 0.80) *−* (26.50 ± 1.00)/(*RT*); R^2^ = 0.999 (1)

*ln k_expACDP_ =* (22.48 ± 0.85) − (22.7 ± 1.00)/(*RT*); R^2^ = 0.999 (2)

Therefore, the *Ea* values are 26.5 ± 1.0 and 22.7 ± 1.0 kcal/mol for DMT and ACDP, respectively, and 1.4 × 10^11^ and 5.8 × 10^9^ s^−1^ for the frequency factors of DMT and ACDP, respectively. In a previous work, *Ea* for ACDP turned out to be 39 ± 2.5 kcal/mol [13], coming from a very different technique, where many reactors (Pyrex vessels) were introduced at a constant temperature (at different temperatures), and the reaction was stopped at different times (up to 50 h) without controlling that all species were in the gas phase. Other cyclic peroxides, such as dioxetane (*Ea* = 22.7 kcal/mol) [11] dimeric diphenyl tetroxane [12] and FDP [7] showed *Ea* = 21.6 and 29.3 kcal/mol, respectively. From these results, it is possible to observe a substitution effect on the *Ea* of this series of compounds. The methyl substitution from FDP to dimethyl tetroxane (DMT) produced a decrease of *Ea*, where the gas phase of DMT is made of a mix of the different position isomers [9]. It is possible to infer that the methyl substitution would go on affecting the *Ea* of these derivatives. *Ea* values for these three derivatives (FDP, DMT, and ACDP) are fitted as a function of the number of methyl groups (*n*), resulting in the following linear function:

*Ea* = (29.28 ± 0.04) − (1.37 ± 0.01)*n*; R^2^ = 0.99 (3)

This indicates an average decrease of 1.4 kcal/molCH_3_ for *Ea*. The average value of the frequency factor of FDP was 5.2 × 10^13^ s^−1^. Our frequency factor shows a much smaller value, having a parallelism with the methyl substitution of the dioxetanes [14,15], which were in the range of 2 × 10^12^–1.3 × 10^13^ s^−1^. Nonetheless, a new linear relationship is also found with the number of methyl group:

*lnA* = (31.10 ± 1.1) − (2.3 ± 0.4)*n*; R^2^ = 0.97 (4)

Therefore, we have to emphasize the additivity of the methyl groups in the thermolysis reaction of the methyl tetroxane derivatives, which could bring further to think that the additivity of the methyl groups in the properties of these compounds would be strongly feasible. The hyperconjugative effect of methyl groups increases the delocalization of the electronic cloud stabilizing the transition states and decreasing the activation energy.

Previous experimental and theoretical works [7,8,9] indicated a stepwise mechanism. In this case, we are going to undertake a computational study in order to confirm and widen the previous proposed mechanisms. 

### 2.3. Thermolysis Reaction Mechanism

**Scheme 1:**
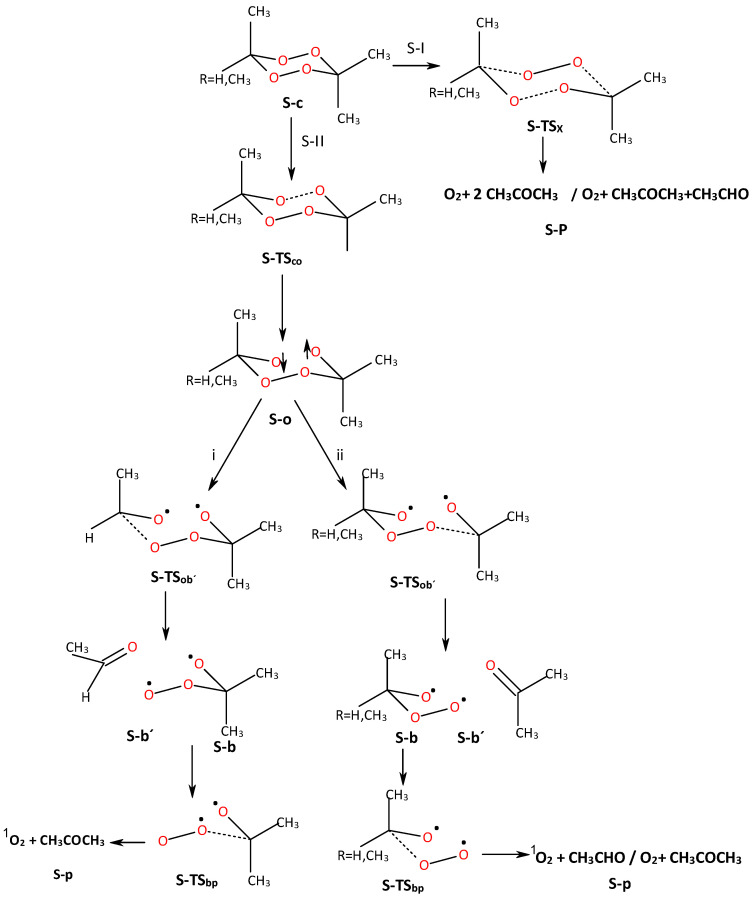
Reaction paths of the singlet ground state ACDP and TMT by single-step/concerted (Branch S-I: from S-**c** to S-**P**) and stepwise (Branch S-II: from S-**c** to S-**p**) mechanisms. Arrow up and down means spin up and down, respectively.

The possible reaction mechanisms are given in Scheme 1 and analyzed with our calculations, in which we can describe a concerted mechanism (S-I) and a stepwise mechanism (S-II) via a diradical open **^·^**O···O**^·^** structure.

#### 2.3.1. Concerted Mechanism

**Figure 1:**
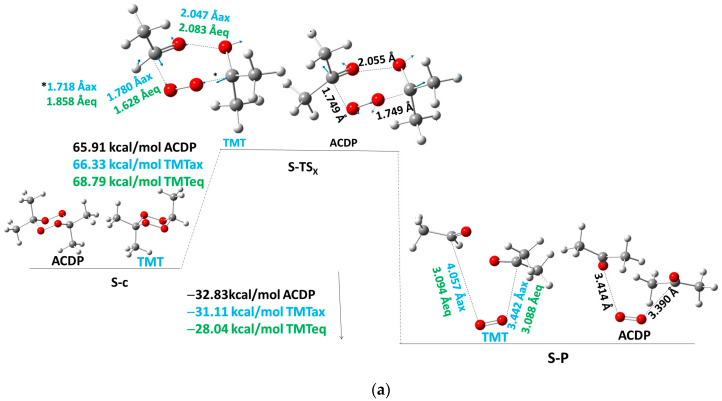

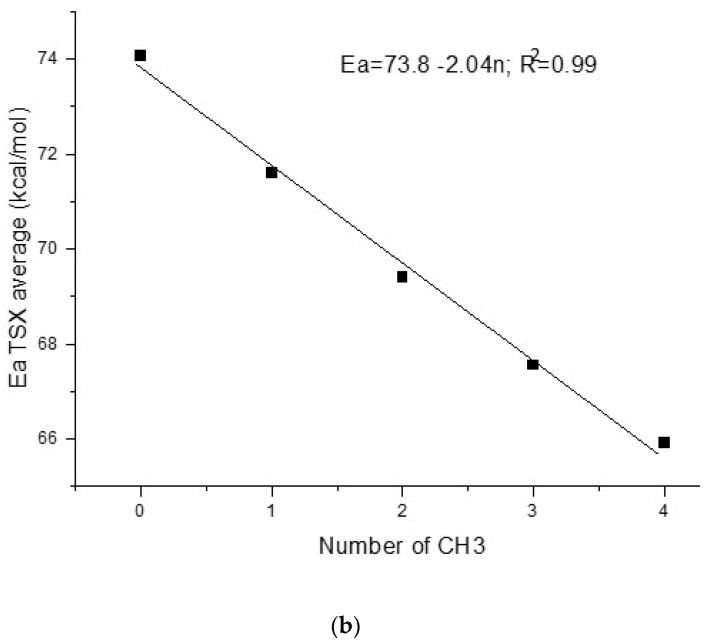
(**a**) Concerted mechanism of S-PES for TMT and ACDP, black color for ACDP, blue for axial TMT, and green for equatorial TMT, and blue arrows indicate the transition vector of TS. (**b**) Activation energy of TS of the concerted mechanism (TS_X_) as a function of the number of methyl groups in FDP. Values for FDP, MFDP, and DMT come from references [7,8,9].

The tri/tetra-methyl-substituted-tetroxane reactants are in a chair conformation (S-**c**). The H atoms of the methyl groups in geminal position show a symmetric eclipsed conformation two to two with respect to the average plane of the molecule, in which all carbons are included (Figure 1a). One H atom of each methyl group is in this plane, and two H atoms out of the plane of two geminal methyl groups are facing each other. The concerted mechanism has been calculated as one single step, where the transition state (TS) is called S-TS_X_ (Table 2, Figure 1a), with a transition vector (TV) of opening and closing the peroxide O-O bonds and opening C···O bonds to form the O_2_ molecule and two molecules of acetaldehyde/acetone via the opening/closing of the C-O bonds. This TS and TV are depicted in Figure 1a. The *Ea* values are 66.3, 68.8, and 65.9 kcal/mol for TMT-axial (TMTax), TMT-equatorial (TMTeq), and ACDP, respectively, being lower than the less-substituted compounds of the series [7,8,9]. The TMTax has lower *Ea* than TMTeq probably due to the anomeric effect that stabilizes the axial isomer TS. 

Taking into account all methyl derivatives, a correlation of the average values of *Ea* as a function of the number of methyl groups has been performed (Figure 1b). We observe that the *Ea* decreases linearly with the increasing number of methyl groups, with a slope of −2.04 kcal/molCH_3_ (Figure 1b; in this figure, average values of axial and equatorial isomers of TMT are also considered). This slope is larger than that of the experimental *Ea* as a function of the number of methyl group (Equation (3)). This fitting shows that the FDP *Ea* for a concerted mechanism would be 73.8 kcal/mol, being considerably higher than the experimental value (29.3 kcal/mol) [7]. Additionally, the value of *Ea* = 65.9 kcal/mol for ACDP is the lowest of the series, which is much higher than our experimental value but is still high enough to avoid the concerted mechanism in front of the stepwise mechanism (vide supra). 

The reaction product (S-P) (Branch S-I, Scheme 1) shows an O_2_ molecule in the singlet state surrounded by hydrogen bonds of two acetaldehyde/acetone molecules depending on if the reactant is TMT or ACDP. Reaction energy (*Er*) is exothermic, being −31.1 and −28.0 kcal/mol for TMT axial and equatorial isomers, respectively, and −32.8 kcal/mol for ACDP. The series follows a linear equation as a function of the number of methyl groups: *Er* = −17.1 − 4.0*n* (R^2^ = 0.98) (FDP is not included in this equation).

#### 2.3.2. Stepwise Mechanism in the Singlet State

The stepwise mechanism is depicted in Scheme 1 (Branch S-II). The mechanism has three steps: (i) the production of the open diradical structure (S-**o**), (ii) the formation of acetone/acetaldehyde (S-**b′**) and oxide-peroxide intermediates (S-**b**), and (iii) the generation of the end products (S-**p**), where the oxygen molecule is in the singlet state. Step (i) is common to all methyl derivatives of tetroxane. In Step (ii), only one possible reaction path is possible for ACDP, but in the case of TMT, two different secondary reaction pathways are possible (vide supra). 

**Figure 2:**
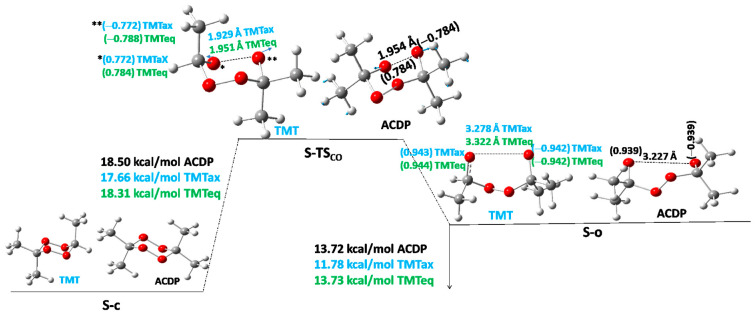
First step of stepwise mechanism S-PES of TMT and ACDP. Black color for ACDP, blue for axial TMT, and green for equatorial TMT. The main non-bonding distances are shown in Å. The spin density is indicated in brackets. Blue arrows indicate the transition vector of TS.

Let us start with the reactant, S-**c**, which goes throughout one TS (S-TS**_co_**) to the open diradical structure S-**o** (Figure 2). This S-TS**_co_** has a much lower *Ea* than the above S-TS_X_. The *Ea* for S-TS**_co_** of TMT turns out to be 17.7 and 18.3 kcal/mol for axial and equatorial methyl position isomers, respectively (Table 3 and Table 4); and for ACDP *Ea* = 18.5 kcal/mol (Figure 2, Table 5). Considering the *Ea* as an average value (*Ea_aver_*) of the axial and equatorial isomers, a function with the number (*n*) of methyl groups of the different derivatives of the series can be expressed by a linear equation *Ea_aver_* = 16.15 + 0.61*n*, R^2^ = 0.993. The most important distance in the TS, and so the simplified reaction coordinate, in the structure of S-TS**_co_**, is the O···O distance, which, in the average of the axial and equatorial isomers (*d_O···Oaver_*), also shows a linear function with the number of methyl groups: *d_O···Oaver_* = 1.90 + 0.014*n*, R^2^ = 0.96. Indeed, the *Ea* and *d*_O···O_ as a function of the number methyl groups show qualitative parallel trends, increasing the distance as increasing *Ea*, but with a much lower slope of 0.014 Å/CH_3_, indicating that the main coordinate of the TS has a small variation on the effect of substituents in the same reaction. 

The first intermediate in the reaction is the diradical open structure, S-**o**, with a distance between both O atoms of 3.22 Å and a spin density of −0.94 and 0.94 e^−^ (Figure 2), indicating that both electrons have different spins, maintaining the identity of the singlet state. This intermediate gives endothermic reaction energy, increasing with the number (*n*) of methyl groups, following also a linear function: *Er***_co_** = 9.0 + 1.22*n*; R^2^ = 0.992. The slope of *Er***_co_** is twice the slope of *Ea*-TS**_co_**, indicating that the methyl substitution effect is twice as large in the diradical open intermediates as in TS**_co_**. Indeed, the linear equation relating both *Er***_co_** and *Ea* (TS**_co_**) is *Er***_co_** = −23.5 + 2.0*Ea* (R^2^ = 0.996). The O···O distance of S-**o** follows the linear equation *d*_O···O_ = 3.519 − 0.074*n*, R^2^ = 0.997. Additionally, the reaction coordinate O···O of S-**o** intermediate decreases with a slope of 4.6 Å with respect to this O···O distance in S-TS**co**, following the linear equation *d*_O···O**o**_ = 12.2 − 4.6*d*(O···O)_TS**co**_ (R^2^ = 0.99). All these effects agree with the Leffler–Hammond postulate [16,17].

**Figure 3:**
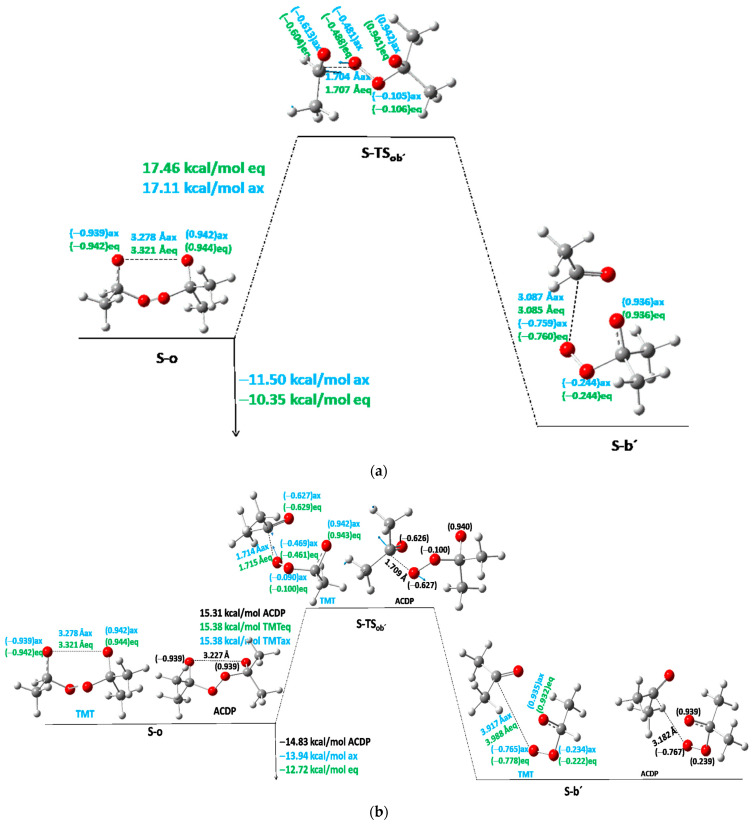
Second step of the stepwise mechanism: (**a**) when acetaldehyde is the first product for both axial and equatorial isomers of TMT; (**b**) for ACDP and for TMT when acetone is the first product for axial and equatorial isomers. The interatomic distance of the breaking bonds are shown, and the spin densities are in brackets. The values of ACDP are in black, and those of TMT are in blue for axial TMT and green for equatorial TMT. Blue arrows mean the transition vector of TS.

The next step is the TS of a broken C-O bond, S-TS**_ob′_**, which depends on either the two-methyl- or single-methyl-substituted C atom at TMT (Figure 3). In the first case, acetone will be obtained as the product (S-**b′,** Branch SII-ii of Scheme 1), while in the second case, the mono-substituted carbon (R = H, CH_3_ in Scheme 1) will give acetaldehyde as the first product of reaction (S-**b′**, Branch SII-i of Scheme 1). Let us start with the mono-substituted carbon (Branch SII-i, Scheme 1). In Figure 3a the S-**o** intermediate goes throughout S-TS**_ob′_** to S-**b′** (second intermediate product). The *Ea* of S-TS**_ob′_** is 17.1 and 17.5 kcal/mol for axial and equatorial isomers with respect to the S-**o** intermediate. In S-TS**_ob′_**, the distinction between equatorial and axial isomers of CH_3_ positions is not clear. Nonetheless, some slightly structural differences are found in the potential energy surface (PES) critical points. For this reason, average values are also taken. The transition vectors are mainly formed by the vibrations of C···O distance—which are 1.704 and 1.707 Å for axial and equatorial isomers, respectively—and the spin density is shared by all oxygen atoms. The product is an acetaldehyde molecule plus an oxide–peroxide diradical, S-**b′** (Figure 3), whose *Er* is exothermic with respect to the S-**o** intermediate. However, it is slightly endothermic with respect to S-**c**. 

When acetone is left as the first product in TMT (Branch ii in Scheme 1), the corresponding *Ea* of TS**_ob′_** and *Er***_ob′_** are 15.4 and −13.9 kcal/mol, respectively, for the axial isomer and 15.4 and −12.7 kcal/mol, respectively, for the equatorial isomer (Figure 3b). This reaction is more favorable than the above, where the aldehyde is left out, though the differences are not meaningful. ACDP yields acetone and a peroxyacetone diradical as reaction products in the second stage, giving 15.31 and −14.83 kcal/mol for *Ea* and *Er*, respectively.

Considering the *Ea* as an average value (*Ea_aver_*) of the axial and equatorial isomers and the two possible intermediates formed in this step of the different derivatives of the series, a linear function can be obtained. These *Ea* values low down to 1.3 kcal/molCH_3_, following the linear equation *Ea*(TS**_o__b′_**) = 20.6 − 1.3*n* (R^2^ = 0.93). The C···O distances have a linear function with respect to the number (*n*) of methyl groups: *d*_(C···O)_ = 1.714 − 0.0013*n* (R^2^ = 0.99). The slope is quite small, indicating that this TS is quite constant with respect to the number of methyl groups. The average values of axial and equatorial isomers give an exothermicity of 2.5 kcal/molCH_3_ with respect to S-**o**, resulting from the linear equation *Er***_ob′_** = −5.0 − 2.5*n* (R^2^ = 0.98). The correlation is low because the product’s disposition is not very regular. In general, the *Ea* of this reaction step decreases with the number of methyl groups, and *Er* is growing in exothermicity. These facts indicate the additivity of methyl groups in our methyl-tetroxane derivatives; that is, each methyl group contributes to decreasing the activation energy.

**Figure 4:**
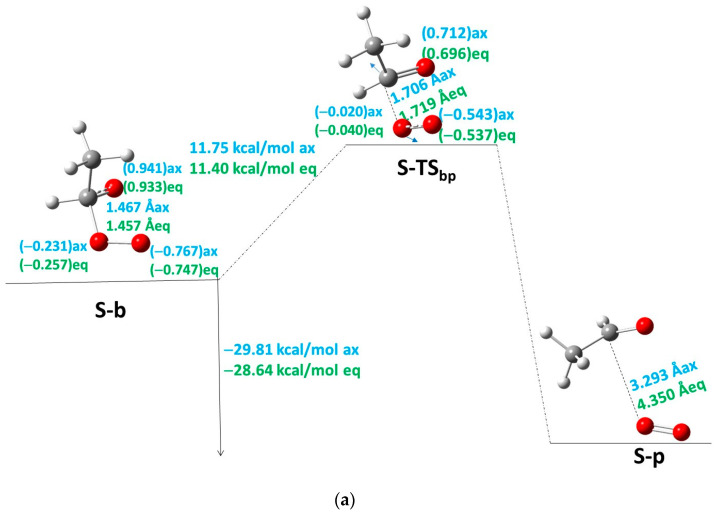

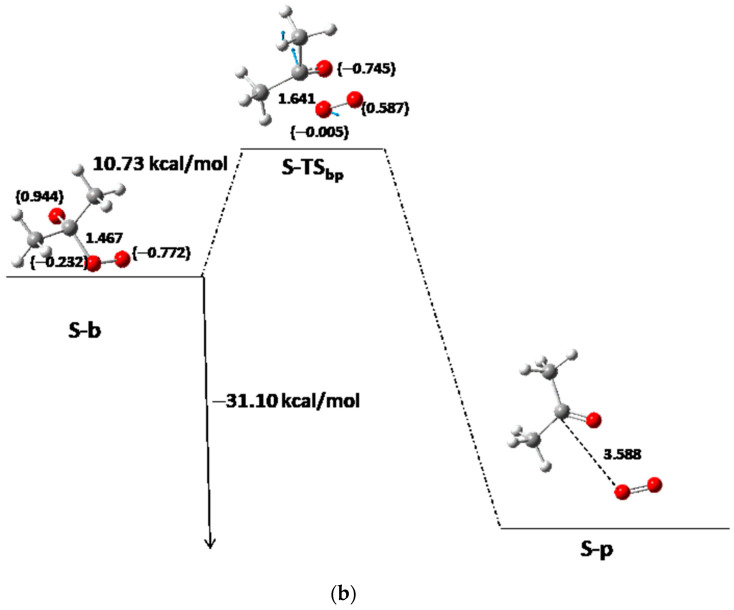
Third step between S-**b** to S-**p** of the S-PES: (**a**) for TMT for axial and equatorial isomers when acetaldehyde and oxygen are the last products; (**b**) for ACDP and for TMT for axial and equatorial isomers when acetone and oxygen are the last products. The interatomic distance of the breaking bond is indicated in Å, and the electron densities are shown in brackets. Black color for ACDP, blue for axial TMT, and green for equatorial TMT. Blue arrows mean the transition vector of TS.

From now on, one of the products of the step (either acetaldehyde or acetone), S-**b′**, is removed, and the oxide–peroxide diradical intermediate (S-**b**) continues reacting. A second molecule of acetaldehyde or acetone and one oxygen molecule will be the products of this reaction step (Table 3, Table 4 and Table 5). When S-**b** is **·**O-CH(CH_3_)-O-O**·,** TMT has the same *Ea* of 11.6 kcal/mol (as average value). This value is similar to MFDP, and DMT [8,9]. The *Er* of the acetaldehyde product (Scheme 1, Branch SII-ii) is −29.01 and −28.64 kcal/mol with respect to **b** for TMTax and TMTeq, respectively (Figure 4a, Table 4). However, when the acetone is the second product, S-**b** is **·**O-C(CH_3_)_2_-O-O**·** (Scheme 1, Branch SII-i), where the *Ea* and *Er* are 10.73 and −31.10 kcal/mol, respectively, for TMT and ACDP (Figure 4b). Therefore, the reaction of TMT and ACDP in the singlet state is exothermic in the last step of reaction. 

In the derivatives with three and four methyl groups, the largest TS is that of the first step—that is, S-TS**_co_**, which could be the rate-limiting step. On the contrary, in FDP, MFDP, and DMT, the rate-limiting step looks like the second step, where S-TS**_ob′_** has the highest energy barrier [7,8,9]. However, these energy differences are very small and both steps can be considered limiting step at experimental conditions. Our experimental *Ea* for ACDP obtained in this work is 22.7 kcal/mol, which is consistent with our theoretical value of 18.5 kcal/mol of S-TS**_co_** (Table 5). 

#### 2.3.3. Thermolysis Reaction as Triplet State (Scheme 2)

For ACDP, a quasi-chair excited structure (T-**qc**) in the PES of the triplet state has been found at 14.9 kcal/mol above S-**c**. This structure is not as symmetric as S-**c** and has a *d*_O···O_ = 2.82 Å, much larger than the other peroxide bonds (1.41 Å). The methyl groups are in the same conformation as S-**c**. This excited structure has a spin density of 0.942 e^−^ on the two open oxygen atoms. The equivalent structure for TMT has not been found. The second structure found in the T-PES is the open diradical structure (T-**o**) close to S-**o**. This T-**o** presents an energy of 13.7 kcal/mol with respect to S-c and only −1.2 kcal/mol with respect to T-**qc**. Then, the energy of T-**o** is very close to T-**qc**, where the quasi-open structure of T-**qc** has a large *d*_O···O_ distance similar to the open structure T-**o**. If a transition state exists from T-**qc** to T-**o**, we could not find it, but it should be with low *Ea* from **T-o** to T-**qc**. 

**Figure 5:**
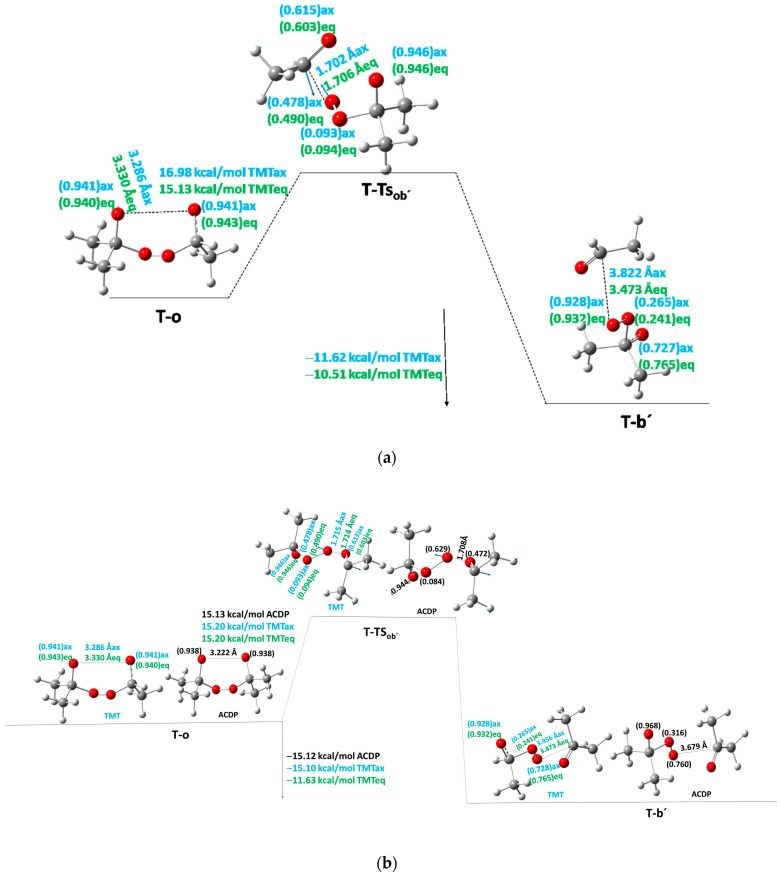
The first steps of the stepwise mechanism between T-**o** and T-**b′** of the T-PES: (**a**) TMT when acetaldehyde is the first product for both axial (ax) and equatorial (eq) isomers; (**b**) TMT and ACDP when the acetone is the first product. Black color is for ACDP, blue for axial TMT, and green for equatorial TMT. Blue arrows indicate the transition vector of TS, the breaking bond distances are indicated in Å, and the spin density values are in brackets.

The energy and structure of T-**o** are very close to the S-**o**, having a spin density of 0.939 e^−^ on each open oxygen of the peroxide bond, and an O···O distance of 3.222 Å for ACDP, and 3.286 and 3.330 Å for TMT axial and equatorial isomers, respectively (Figure 5). The O···O distances decrease as the number (*n*) of methyl groups following the linear equation *d*_O···O_ = 3.527 − 0.075*n* (R^2^ = 0.998). This equation and distances are very close to the S-**o**-*d*_O···O_ distances, indicating the similar **o** structures for the different derivatives and their very close variation with the number of methyl groups. 

**Scheme 2:**
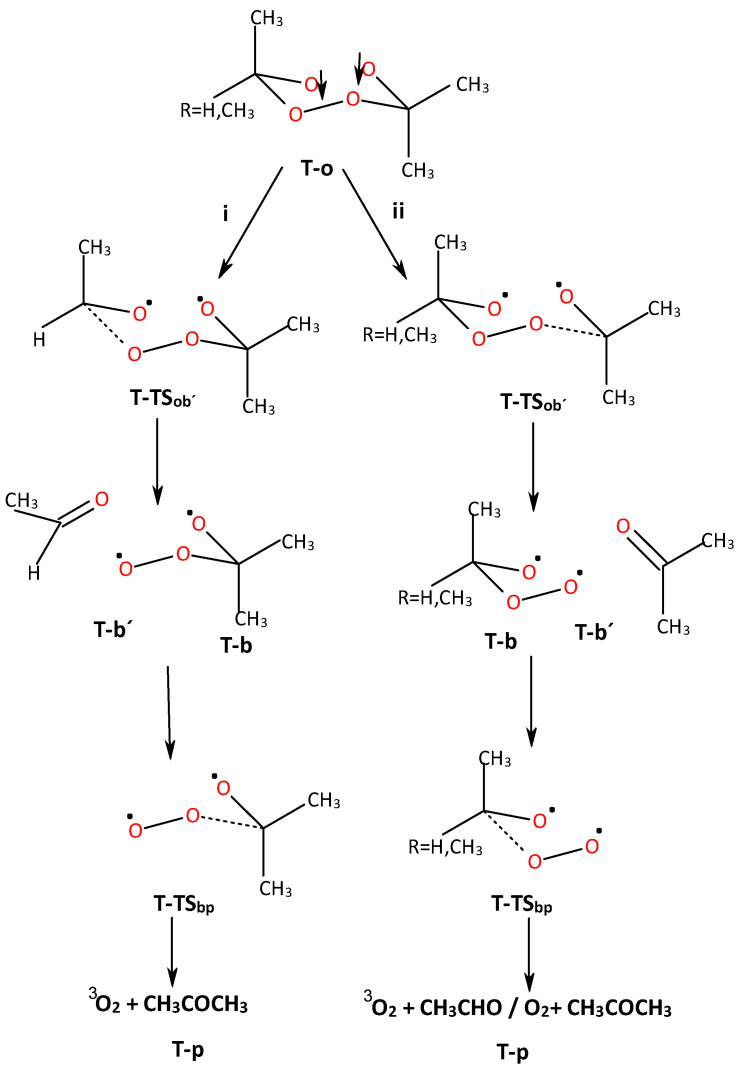
Reaction paths of the triplet ground states of ACDP and TMT. The two arrows down means two parallel spins.

From T-**o** a transition state T-TS**_ob′_** is also found. This new structure is the TS for breaking and forming the first acetone/acetaldehyde molecule. In TMT, different values are found depending on if either acetone or acetaldehyde is the first product in **b′**. If acetaldehyde is the first product in **b′** (Scheme 2, Branch i), *Ea* is 16.98 and 15.13 kcal/mol for the axial and equatorial isomers, respectively (Figure 5a). If acetone is the first product (T-**b′**, Scheme 2, Branch ii), *Ea* is 15.20 kcal/mol for both axial and equatorial isomers (Figure 5b). In ACDP, *Ea* is 15.13 kcal/mol with respect to T-**o** (Figure 5b). The *Ea* as a function of the number of methyl groups from FDP to DMT was previously calculated with a quadratic function [9]. With the addition of the three and four substituted derivatives to the system, the quadratic function is *Ea* = 25.3 − 6.2*n* + 0.9*n*^2^ (R^2^ = 0.97), similar to that found in Reference [9] (Table 6, Table 7 and Table 8).

C···O distances for T-TS**_ob′_** are 1.708 Å for ACDP and 1.704 and 1.715 Å for TMT as average values of the position axial/equatorial isomers when acetaldehyde and acetone are the first products, respectively. These distances are not quite different from those of S-TS**_ob′._** TV (Figure 5) is viewed as a vibration of the C···O breaking/forming the C···O bond. These distances vary with the number (*n*) of methyl groups with a linear function: d_C···O_ = 1.714 − 0.0014*n* (R^2^ = 0.991). The slope of the function is very small, where the structure of the TS can be considered very stable as the number of methyl groups increases. This equation is very close to the d_C···O_ equation of the S-TS**_ob′_**, which indicates an identity of structures in the TS**_ob′_** of the diradical part of reaction independently of the multiplicity of the state. Additionally, the **o** diradicals also have very close structures for both multiplicity states, as already mentioned above. From this, we can extract that if both multiplicity states have equal reactants and TS’, the reaction path should be very close. The spin density of T-TS**_ob′_** is shared over all oxygen atoms in the structure (Figure 5). 

**Figure 6:**
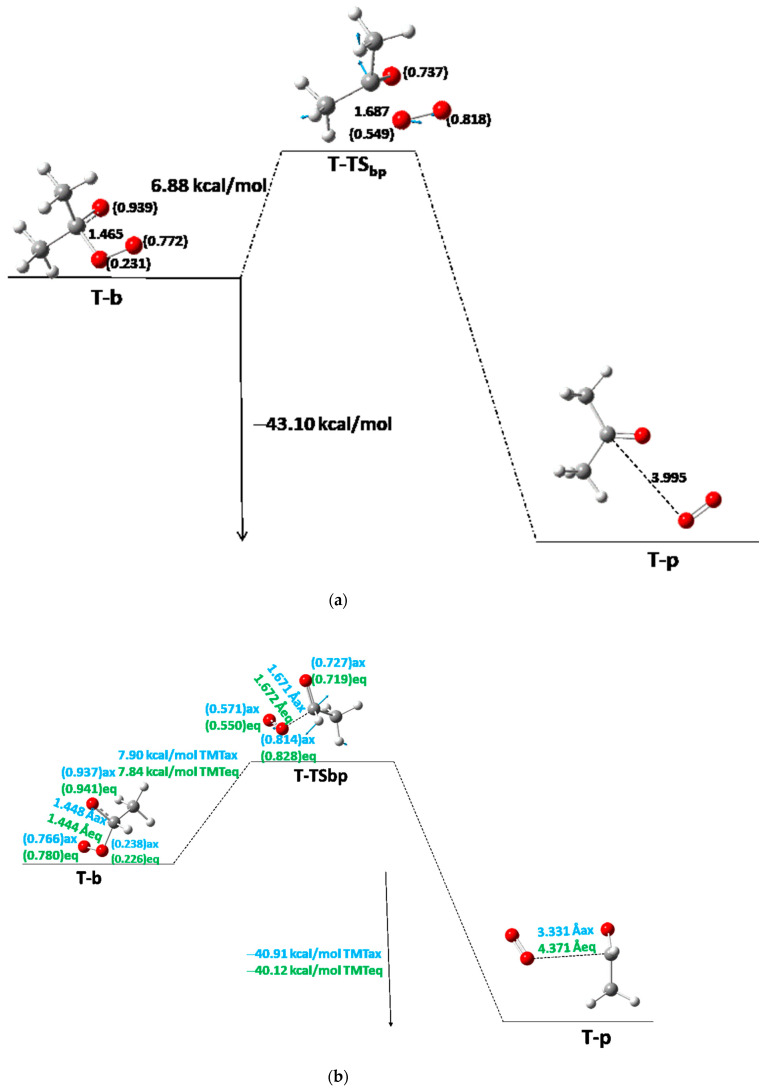
The second step between T-**b** to T-**p** of the T-PES of (**a**) TMT and ACDP when the acetone and oxygen are the products; (**b**) TMT when the acetaldehyde and oxygen are the products. The distance of the breaking bond is indicated in Å, and the spin density values are in brackets. The TVs are indicated by blue arrows.

T-TS**_ob′_** goes to T-**b′**, a super-molecular system with an acetone/acetaldehyde molecule and a peroxy-acetaldehyde/acetone diradical, with a C_acetone/acetaldehyde_···O_peroxy_ distance of 3.956 and 3.473 Å for TMT_aver(ax-eq)_ and acetone as the first product of the reaction; 3.647 Å is the average of both position isomers when the acetaldehyde is the first product of the reaction. The reaction energy is −11.06 kcal/mol with respect to T-**o** for TMT as an average value of both position isomers when acetaldehyde is the first product of reaction (Figure 5a, Table 6); and −13.36 kcal/mol for the equivalent value when acetone is the first product of the reaction (Figure 5b, Table 7). The *Er* as a function of the number of methyl groups behaves decreasing with the linear function *Er* = −5.5 − 2.9*n*, R^2^ = 0.93. The spin density of the triplet state is shared on the three oxygen atoms of the peroxy-acetone radical. From now on, the acetone molecule is removed from the super-molecular system and the critical point is optimized from the peroxy-acetone radical. Nonetheless, the energy of acetone molecule is constantly added up to the energy of the critical points in order to preserve the whole energy of the system. Following this diradical intermediate, T-**b**, a T-TS**_bp_** yields the final product (T-**p**, Figure 6). The *Ea* is really the lowest of the T-PES and S-PES with values of 6.88–7.90 kcal/mol (Table 6, Table 7 and Table 8). The *Ea* as a function of the number of methyl groups is also linear: *Ea* = 9.48 − 0.67*n*, R^2^ = 0.98. In the T-PES all *Ea*s low down with the increasing number of methyl groups, indicating the reaction rate is faster with the methyl substitution. Besides, this TS**_bp_** is the lowest TS of the S- and T-PES. The last TS gives rise to the second acetaldehyde/acetone molecule plus the ^3^O_2_ molecule. The TVs clearly show this formation (Figure 6a,b). The spin density also is shared over the three oxygen atoms of the system. The highest *Ea* on T-PES is that of T-TS**_ob′_**, being the rate-limiting step of the reaction, which agrees with the other derivatives of the series.

Finally, the products are ^3^O_2_ plus the second acetone/acetaldehyde molecule. The reaction energy with respect to T-**o** is exothermic, with −58.2 kcal/mol for ACDP (*Er***_ob′_**+ *Er***_bp_**, Table 8) and −54.2 and −53.9 kcal/mol for TMT_aver(ax-eq)_ for acetone (Table 6) and acetaldehyde (Table 7) as the second product, respectively. A linear function is also found for this step of reaction, *Er***_op_** = −41.4 − 4.3*n*, R^2^ = 0.998, describing the reaction energy of **op**, giving an exothermicity of 4.3 kcal/molCH_3_. Considering the first step of the reaction in singlet state for obtaining the biradical S-**o**, the reaction energies of the reactionin triplet state with respect to reactant S-**c** are −44.7 kcal/mol for ACDP and −41.4 and −40.7 kcal/mol for TMT_aver(ax-eq)_ for acetone and acetaldehyde as final products, respectively. If the reaction would proceed in singlet state PES, the *Er* of the equivalent critical point for S-**p**, with respect to S-**c** is −24.0 kcal/mol and −29.9 kcal/mol for TMT_aver(ax-eq)_ for acetone and acetaldehyde as final products, respectively and −32.4 kcal/mol with respect to S-**c** for ACDP (Table 3, Table 4 and Table 5). This indicates that the reaction energies on T-PES are much more exothermic than in the S-PES, as expected based on the fact that the singlet molecular oxygen is more unstable than triplet molecular oxygen. The results in the T-PES are more in agreement with the experimental reaction energies of the diperoxide compounds.

The influence of the methyl groups in the thermolysis reaction of tetroxane derivatives increases the exothermicity of the reaction, and they change the rate-limiting step from the second step to the first step, although the differences in Ea are very small, and both TSs could be viewed as limiting steps at experimental conditions; additionally, they also change the geometry of intermediates and transition states in an approximately linear way. The singlet and triplet states present very close structures at the different critical point intermediates of the reaction. These similarities of structures indicate a possible nonadiabatic transition from the singlet to triplet states in such a way that reaction would be more exothermic if this transition of different multiplicity states is produced, which would agree with the high exothermicity of the tetroxanes. Therefore, it is worth providing general details on the intersystem crossing process, as described in the next section. 

### 2.4. Excited States and Non-Adiabatic Chemistry of Tetroxane and the Methylated Systems

**Figure 7:**
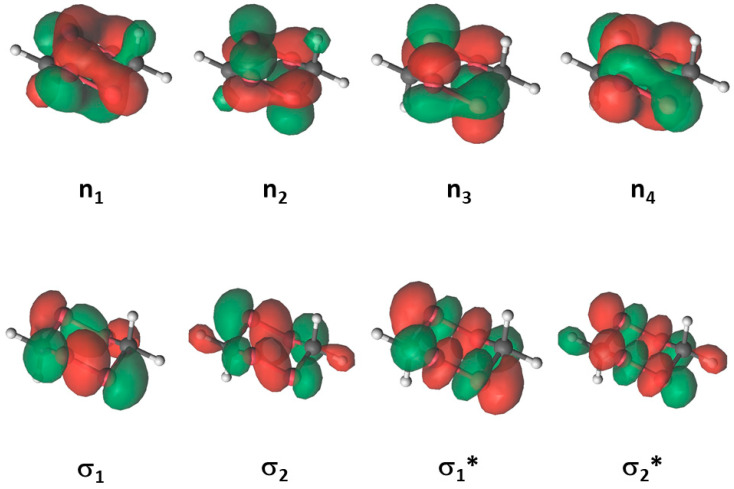
Bonding (green color) and anti-bonding (* and red color) natural orbitals used in the CASSCF/CASPT2(12-in-8)/ANO-L-VDZP computations of FDP.

The lowest-lying singlet and triplet excited states of tetroxane (FDP) at the reactant structure correspond to electronic excitations from the lone pairs of the oxygen atoms (n*_i_*) or the *σ* bonding orbitals of the -O-O- bonds (*σ_i_*) to the *σ* anti-bonding orbitals of those bonds (σ^*^_j_)—that is, n*_i_* → σ^*^_j_ or *σ_i_* → σ^*^_j_ excitations (see orbitals in Figure 7). These electronic configurations are associated with the weakening of the peroxide bonds. The vertical energy position of the three first singlet (S_i_) excited states (all of them n*_i_* → σ^*^_j_ in nature) relative to the energy of the ground state (S_0_) ranges from 5.09 to 5.78 eV (Table 9). The manifold of triplet states (also n*_i_* → σ^*^_j_ in nature) is lower-lying than that of the singlet states, being in a range of 3.87–4.81 eV for the first four of them. Test computations show that *σ_i_* → σ^*^_j_ configurations appear on the geometry of the reactant at higher energies (Table 9).

**Figure 8:**
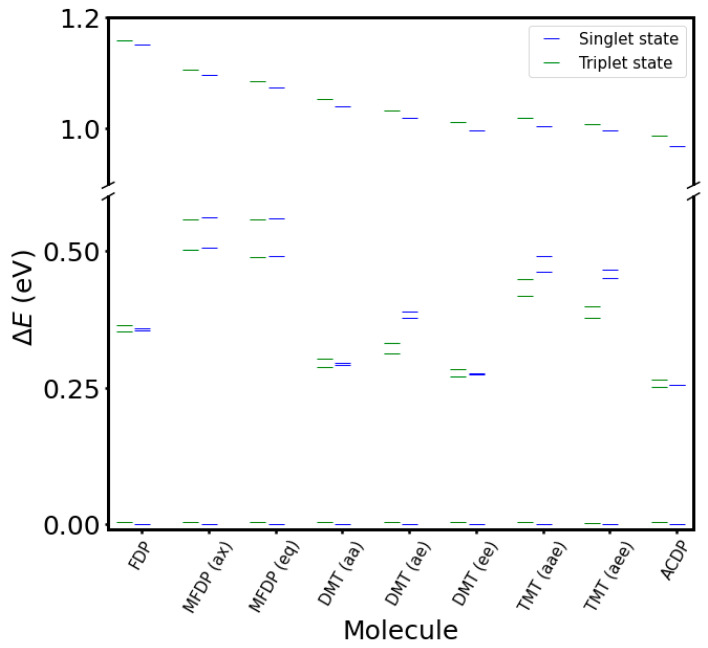
Vertical energies of the four singlet and four triplet electronic states at the ground-state geometry of the diradical obtained with the CASSCF/CASPT2(12-in-8)/ANO-L-VDZP calculations. Green levels are excited triplet states (from T_1_ to T_4_), and blue levels are ground singlet state (S_0_) and their excited states (S_1_ to S_3_) of each derivative in increasing order of energy.

Upon -O-O- bond stretching, the mentioned electronic configurations become stabilized as is confirmed by the calculated energy of the excited S_i_ and T_i_ states at the diradical geometry. Here, the four singlets and four triplet states appear in an energy range lower than 1 eV (Figure 8). T_1_ has the same energy as the ground state (S_0_), and other states (S_1_, S_2_, T_2_, and T_3_) are located only 0.3 eV above S_0_. Considering that the kinetic energy released after the transition state of the -O-O- bond breaking is of the same order; those excited states may be populated in the non-adiabatic dynamical process, which follows the peroxide bond decomposition.

To analyze the efficiency of the non-adiabatic transitions at the diradical region, SOCs were calculated between the different singlet and triple states (Table 10). The coupling between S_0_ and T_1_ is negligible, and the maximum value is obtained with T_3_, which is located at 0.29 eV. Additionally, a spin orbital coupling (SOC) between S_2_ and T_1_ at 0.29 eV also has a very high value. Both SOCs indicate two possibilities for transferring the population from S_0_ to T_1_ along the thermal decomposition: (i) S_0_ transfers the population initially to T_3_ via strong SOC and subsequently by an ultrafast internal conversion process T_3_ decays to T_1_, or (ii) S_0_ transfers the population initially to S_2_ via an internal conversion process and next the molecule changes to the T_1_ manifold through strong SOC between S_2_ and T_1_.

**Figure 9:**
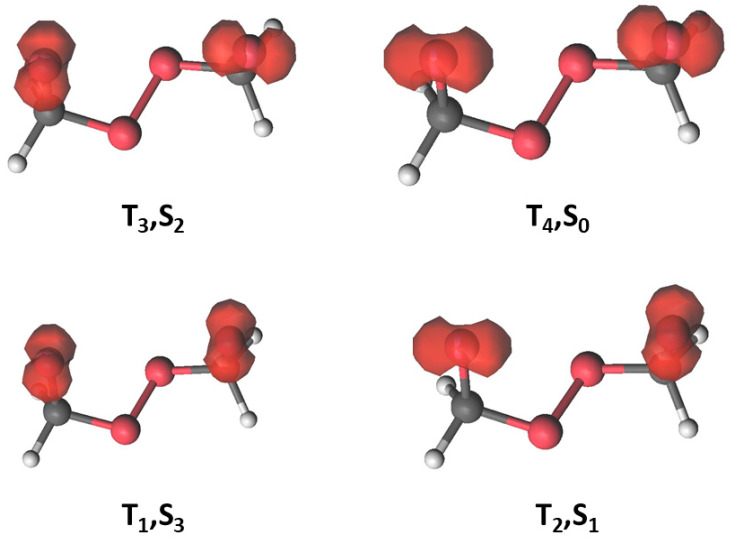
CASSCF (12-in-8)/ANO-L-VDZP electron spin density for the triplet states of the diradical of FDP. The corresponding densities for the singlets S_0_, S_1_, S_2_, and S_3_ are similar to those for T_4_, T_2_, T_3_, and T_1_, respectively.

Analysis of the open-shell spin densities at the diradical region shall help to provide a rationale on the distinct efficiencies related to the population transfer between the singlet and triplet states (Figure 9). Such densities reflect the distinct occupation of the unpaired electrons of the diradical in the oxygen 2*p_x_* and 2*p_y_* atomic-like orbitals, which are perpendicular to the C-O bonds. As can be seen, high SOC values are obtained when singlets and triplets have one unpaired electron in a perpendicular 2*p* orbital, in accordance with El-Sayed’s rules [18].

Once we comprehend the electronic-structure features of the parent tetraoxane molecule (FDP), it is worth extending the analysis to the methylated molecules of the family, Figure 8 shows that ACDP has the electronic states closer in energy compared with FDP. Energies of S_3_ and T_4_ show a clear trend of decreasing energy upon methylation. Regarding the most accessible states (S_1,_ S_2_, S_3_, T_2_, and T_3_), we see that an odd number of methyl group substitutions (MFDP and TMT) increases the energy gap, while those states are energetically slightly more accessible for FDP and the molecules with an even number of methyl groups. Comparison of SOCs in the distinct molecules of the family (Table 10) also do not show an efficient transition from S_0_ to T_1_ at the diradical region and a higher efficiency for S_0_ -> T_3_ and S_2_ -> T_1_. The highest values appear also for the molecules with an even methylation. ACDP, which shows the lowest energy position of the S_2_ and T_3_ states at the diradical region and high SOCs arises as the most efficient system in the family for non-adiabatic decomposition. The dynamical effects, not considered in this work, are expected to favor this triplet population even more, as inferred from the study by Vacher et al. comparing the dynamics of 1,2-dioxetane and the tetramethylated compound [19]. By increasing the mass of the substituents, the time spend at the diradical region increases, allowing a more effective re-distribution of the population among the near-degenerate singlet and triplet states and accordingly higher yields for the evolution on T_1_ [19]. These transitions are more favorable for FDP and even-methylated derivatives (the highest SOCs and the least ΔE), being less efficient at the odd-methylated compounds. For FDP and the even-methylated derivatives, SOC is linear with the number of methyl groups (SOC_S2->T1_ = 96.15–0.96·*n*; R^2^ = 0.999; SOC of DMT being the average of the three position isomers); SOC of S_0_ to T_3_ equally decreases with the number of methyl groups, but non-linearly. ΔE (Figure 8) of the tetramethyl derivative is the least of all derivatives. SOCs of both transitions of the two odd-methylated derivatives increase with the number of methyl groups, SOCs at S_2_ to T_1_ being the highest.

## 3. Materials and Methodology

### 3.1. Materials

DMT was prepared by reacting 0.6 mL of acetaldehyde, which is added to a stirred and cooled solution at 263.15 K containing 2.5 mL of ethanol (absolute; Merck, Darmstadt, Germany), 0.3 mL of hydrogen peroxide (56% *v*/*v*) and 1.5 mL of sulfuric acid (98%, Merck). The mixture was stirred, and sulfuric acid was added up to 3 mL, precipitating a white solid. Then, water was slowly added to complete a volume of 5 mL in order to increase the insolubility of the peroxide precipitate in the alcoholic medium. A white precipitate with a gelatinous characteristic was obtained, which was washed and centrifugated repeatedly with distilled water until neutral pH. Thus, the wet solid was dissolved directly in benzene (avoiding the incorporation into the organic phase of possible polar impurities). This dissolution was analyzed using the gas chromatographic (GC) method by using an Agilent 7890-A chromatograph (Santa Clara, CA, USA).

ACDP was synthesized by carefully adding acetone to a stirring solution of hydrogen peroxide in strong acid medium at 253 K, as it was previously proposed [7]. ACDP solid was characterized by IR spectroscopy by using a Nicolet spectrometer (Thermo Fisher Scientific, Waltham, MA, USA), and a solution was analyzed by UV spectroscopy with a CampSpec M330 spectrophotometer (Leeds, UK) and GC. THF was used as a solvent in the thermolysis reaction of ACDP.

### 3.2. Analytic and Kinetic Methods

The GC method was used to study the kinetic of the thermolysis reaction of DMT and ACDP. This technique is very suitable for small quantities of samples. The reactor in this method is the injection chamber of a gas chromatograph, where the flow and reaction time (40 s) are maintained constant, and the reaction temperature is a parameter. When the compound flows out of the reactor and goes to the chromatographic column, temperature lowers enough to stop the thermolysis reaction, but temperature is maintained high enough to produce the chromatographic separation [12] (ΔT = −150 K in the most adverse case). The method was also used for FDP, obtaining good results [7]. 

A solution (1 μL) of DMT (0.001 M) in benzene was introduced in the injection chamber. The reaction was controlled by the injector temperature within the range of 493.15–543.15 K, and the rate of ΔT = 10 K. As the flow and linear rate were kept constant, the reaction time is consequently kept also constant. The reaction products and reactant were analyzed by GC with an electronic device of constant flow (Q = 1.40 mL/min; *V_L_* = 20 cm/s) using nitrogen as carrier gas. A flame-ionization detector and a melted silica capillary column (25 m × 0.53 mm) coated with 5% of phenyl-methylpolysiloxane were used. Temperature of the chromatographic column was started at 313 K for 2 min, then programmed at a rate of 30 K/min up to 463 K, and held constant for 10 min.

A solution (1 μL) of ACDP (0.02 M) in THF was introduced in the injection chamber under the same experimental conditions as DMT. The reaction was controlled by the injector temperature. 

Residence time, which coincides with the reaction time, in the injector chamber was calculated by Reference [12]:

t = l/v(5)

where *l* (cm) is the liner length and *v* is the linear rate of the carrier gas. All previous works have found the first-order reaction model for this kind of thermolysis reaction [7,12]. Then, *Ea* was calculated by the Arrhenius equation in a first-order integral kinetic equation such as:

ln[ln(C_0_/C)] = −Ea/RT + ln(A·t) (6)

where *C*_0_ and *C* are the initial and remaining concentrations of the reactant, respectively; *T* is the temperature; *A* is the frequency factor of the Arrhenius equation; and *t* is the residence time, Equation (4). *ln*[*ln*(*C*_0_/*C*)] is fitted as a function of 1/*T* to a linear equation.

### 3.3. Computational Methods

The electron density properties of the studied molecules (ACDP and TMT) were calculated according to references [7,8,9]. The geometry and energy of the reactant, intermediates, and products were computed by using the Gaussian 09 program [20], at the DFT/BHANDHLYP/6-311 + G** level [21,22,23]. Critical points (minimums and TS) of the S- and T-PES were calculated independently and optimized with the Berny method [24]. The topology of the critical point was characterized by means of the harmonic frequencies. Transition vectors (TVs) for the TSs were inspected and drawn with the help of the GaussView package [25]. The intrinsic reaction coordinate method was used in order to connect the reaction paths between the TS and reactant, products or intermediates [26,27]. The relative energies of the critical points of the PES were calculated by adding the total energy to the zero-point energy (ZPE).

The tetroxane series (FDP, MFDP, DMT, TMT, and ACDP) with their relevant axial and equatorial conformers, as determined in the current and previous related works, was revisited to obtain a high-level characterization of distinct nonadiabatic transitions between T and S states occurring along the thermal decomposition reaction. Four S states (S_0_–S_3_) and four T states (T_1_–T_4_) were energetically characterized accounting for the spin orbit coupling (SOC) and transition probability between all of them. The CASSCF/CASPT2 method [28,29] as implemented in OpenMolcas [30] was used to compute energies and SOCs at the DFT geometries. The valence double-ζ plus polarization atomic natural orbital L-type basis set (ANO-L-VDZP) was employed. State-average CASSCF wave functions, averaging 4 S and 4 T separately, were used as zeroth-order references for the CASPT2 calculations. The active space comprised 12 electrons distributed in 8 orbitals, CASSCF(12,8). In the CASPT2 computations, the ionization potential electron affinity parameter (IPEA) was set to 0.00 au, and an imaginary level shift of 0.2 au [31] was employed to minimize the presence of weakly intruder states. SOC matrix elements $\left\langle T_{x,m_{s}=-1,0,1}|{\hat{H}}_{SO}|S_{y}\right\rangle$ were computed using the CASSCF wave functions. The SOC complex vector norms $\left|{\hat{H}}_{SO}(T_{x,m_{s}=-1,0,1}/S_{y})\right|$ were obtained as the square root of the sum of the absolute square of each $m_{s}$ component of the triplet state $T_{x}$ with the corresponding singlet state $S_{y}$. The effective one-electron spin–orbit Hamiltonian was derived from atomic mean field integrals, employing the RASSI method [32,33].

## 4. Conclusions

In this work, experimental activation energy and the pre-exponential factor of the thermolysis reaction of DMT and ACDP have been studied by means of gas chromatography. Thermolysis reactions were produced in the injector chamber at different temperatures. A first order velocity constant was found experimentally. The experimental activation energies are 26.5 and 22.7 kcal/mol for DMT and ACDP, respectively. Additionally, the reaction mechanism of this reaction was accomplished by means of theoretical methods in TMT and ACDP. All features of the mechanism were searched as a function of the number of methyl groups. Position isomers were also considered. Finally, the first S and T excited states of the series were also calculated, and the SOC between them was determined.

The calculated *Ea* of the stepwise mechanism was lower than the concerted mechanism, and it agrees with the experimental results of the tetroxane derivatives and other similar compounds. The first intermediate is a ·O···O· diradical structure, which is very close in geometry and energy and has similar structure to the triplet diradical. The next step is the C···O dissociation and the production of the first molecule of acetaldehyde/acetone in the singlet state. The transition state for this dissociation is also very similar to that of T state, and the geometry and energy of the open diradical and the transition states are linear with the number methyl groups. If it would be possible to extrapolate the reaction paths, they would be also very close for both multiplicity states.

The reaction energies of both states are exothermic, but those of the T-PES is much more exothermic. A transition from the S to T state in the open diradical could be produced by two possible mechanisms: (i) from the singlet ground state to S_2_, for internal conversion and from this one to T_1_ by one strong SOC; and (ii) from S_0_ to T_3_ by a strong SOC and from this one to T_1_, decaying for internal conversion. Such singlet to triplet non-adiabatic transition seems to be more hindered for the mono- and tri-methylated tetroxanes, being more favorable in FDP and the di- and tetra-methylated molecules, in particular the last one.

Therefore, our experimental and theoretical studies emphasize the additivity of the methyl groups in the thermolysis reaction of the methyl-tetroxane derivatives and its properties. This work is a clear example where theoretical calculations can explain the experimental results of the behavior of these tetroxanes, the reaction products, and the mechanisms. The knowledge acquired from this work can be applied to obtain better control of these processes at laboratory and industrial scales, with economic and environmental impact in the industry along with better therapeutic applications.

## Figures and Tables

**Table 1:** Temperature (K) and rate constant (*k*) (s^−1^) of DMT 0.001 M in solution of benzene and ACDP 0.02 M in solution of tetrahydrofuran (THF).

*T*	*k_exp,DMT_*	*k_exp,ACPD_*
493	0.245	0.461
503	0.419	0.732
513	0.704	1.142
523	1.072	1.749
533	1.867	2.642
543	2.959	3.926

**Table 2:** Total energies (E) ^a^, zero-point energies (ZPE) ^a^, imaginary frequencies ν ^b^, spin contamination S^2^, and relative energies (ΔE) ^c^ of critical points of the singlet-ground-state PES of the thermolysis reaction of TMT and ACDP. Concerted mechanism.

**Structure**	E ^a^		ZPE ^a^		ν ^b^		S^2^		ΔE ^c^	
TMT ^d^										
	C-6ax	C-6eq	C-6ax	C-6eq	C-6ax	C-6eq	C-6ax	C-6eq	C-6ax	C-6eq
**c**	−497.124296	−497.129205	0.160475	0.160432			0.000	0.000	0.00	0.00
TS**_x_**	−497.010763	−497.011919	0.152643	0.147980	847	782	0.000	0.000	66.33	68.79
**P**	−497.162535	−497.162748	0.149117	0.149286			0.000	0.000	−31.12	−28.04
ACDP										
	E ^a^		ZPE ^a^		ν ^b^		S^2^		ΔE ^c^	
**c**	−536.430463		0.188318				0.000		0.00	
TS**_x_**	−536.318198		0.181089		827		0.000		65.91	
**P**	−536.472492		0.178024				0.000		−32.83	

^a^ Hartree. ^b^ Imaginary frequencies in cm^−1^. ^c^ Relative energies in kcal/mol, $\Delta E=[E_{total}+ZPE](c)-\left[E_{total}+ZPE\right](i)$, where ***i***, represents any critical point of the species in the table. ^d^ In position C-6 axial (ax) and equatorial (eq) isomers.

**Table 3:** Total energies (E) ^a^, zero-point energies (ZPE) ^a^, imaginary frequencies (ν) ^b^, spin contamination (S^2^), and relative energies (ΔE) ^c^ of critical points of the singlet-ground-state PES of the thermolysis reaction of 3,3,6-trimethyl-1,2,4,5-tetroxane (TMT) in position C-6 axial (ax) and equatorial (eq) isomers. Acetaldehyde as the first intermediate product (Figure 3a) and acetone as the second product (Figure 4b).

**Structure**	E ^a^		ZPE ^a^		ν ^b^		S^2^		ΔE ^c^	
	C-6ax	C-6eq	C-6ax	C-6eq	C-6ax	C-6eq	C-6ax	C-6eq	C-6ax	C-6eq
Steps 1–2										
**c**	−497.124296	−497.129205	0.160475	0.160432			0.000	0.000	0.00	0.00
TS**_co_**	−497.092215	−497.096180	0.156538	0.156593	290	235	0.029	0.030	17.66	18.31
**o**	−497.101741	−497.101741	0.154981	0.154855			0.079	0.079	11.78	13.73
TS**_ob′_**	−497.070352	−497.071563	0.152574	0.152500	921	880	0.434	0.438	28.89 (17.11) ^d^	31.19 (17.46) ^d^
**b′**	−497.114959	−497.114956	0.151574	0.151577			0.096	0.096	0.27 (−11.50) ^d^	3.38 (−10.35) ^d^
Step 3										
**b**	−343.323732	−343.323732	0.093465	0.093465			0.093	0.093	0.00	0.00
TS**_bp_**	−343.303619	−343.303619	0.090456	0.090456	1748	1748	0.331	0.331	10.73	10.73
**p**	−343.370545	−343.370545	0.090715	0.090715			0.000	0.000	−31.10	−31.10

^a^ Hartrees. ^b^ Imaginary frequencies in cm^−1^. ^c^ Relative energies in kcal/mol, $\Delta E=[E_{total}+ZPE](c/b)-[E_{total}+ZPE](i)$ where ***i***, represents any critical point of the species in the table. ^d^ Relative energies in kcal/mol with respect critical point **o**.

**Table 4:** Total energies (E) ^a^, zero-point energies (ZPE) ^a^, imaginary frequencies (ν) ^b^, spin contamination (S^2^), and relative energies (ΔE) ^c^ of critical points of the singlet-ground-state PES’ of the thermolysis reaction of 3,3,6-trimethyl-1,2,4,5-tetroxane (TMT) in position C-6 axial (ax) and equatorial (eq) isomers. Acetone as intermediate product of step 2 (Figure 3b) and acetaldehyde as product of the last step (Figure 4a).

**Structure**	E ^a^		ZPE ^a^		ν^b^		S^2^		ΔE ^c^	
	C-6ax	C-6eq	C-6ax	C-6eq	C-6ax	C-6eq	C-6ax	C-6eq	C-6ax	C-6eq
Steps 1–2										
**c**	−497.124296	−497.129205	0.160475	0.160432			0.000	0.000	0.00	0.00
TS**_co_**	−497.092215	−497.096180	0.156538	0.156593	290	235	0.029	0.030	17.66	18.31
**o**	−497.100033	−497.101741	0.154981	0.154855			0.079	0.079	11.78	13.73
TS**_ob′_**	−497.073225	−497.074872	0.152687	0.152499	840	850	0.434	0.429	27.16 (15.38) ^d^	29.11 (15.38) ^d^
**b′**	−497.119271	−497.118808	0.152003	0.151632			0.097	0.095	−2.16 (−13.94) ^d^	1.00 (−12.72) ^d^
Step 3										
**b**	−304.018286	−304.018882	0.065015	0.064871			0.094	0.099	0.00	0.00
TS**_bp_**	−303.996727	−303.997398	0.062188	0.061560	3892	4396	0.377	0.389	11.75	11.40
**p**	−304.061264	−304.061138	0.061760	0.061722			0.000	0.000	−29.01	−28.64

^a^ Hartrees. ^b^ Imaginary frequencies in cm^−1^. ^c^ Relative energies in kcal/mol, $\Delta E=[E_{total}+ZPE](c/b)-[E_{total}+ZPE](i)$, where ***i***, represents any critical point of the species in the table. ^d^ Relative energies in kcal/mol with respect to critical point **o**.

**Table 5:** Total energies (E) ^a^, zero-point energies (ZPE) ^a^, imaginary frequencies (ν) ^b^, spin contamination (S^2^), and relative energies (ΔE) ^c^ of critical points of the singlet-ground-state PES’ of the thermolysis reaction of 3,3,6,6-tetramethyl-1,2,4,5-tetroxane (ACDP).

**Structure**	E ^a^	ZPE ^a^	ν ^b^	S^2^	ΔE ^c^
Step 1					
**c**	−536.430463	0.188318		0.000	0.00
TS**_co_**	−536.397311	0.184653	233	0.031	18.50
**o**	−536.403912	0.183363		0.077	13.55
TS**_ob′_**	−536.377167	0.181022	846	0.422	28.86 (15.31) ^d^
**b′**	−536.424636	0.180440		0.096	−1.29 (−14.83) ^d^
Step 2					
**b**	−343.323732	0.093465		0.093	0.00
TS**_bp_**	−343.303619	0.090456	1748	0.331	10.73
**p**	−343.370545	0.090715		0.000	−31.10

^a^ Hartrees. ^b^ Imaginary frequencies in cm^−1^. ^c^ Relative energies in kcal/mol, $\Delta E=[E_{total}+ZPE](c/b)-[E_{total}+ZPE](i)$, where ***i***, represents any critical point of the species in the table. ^d^ Relative energies in kcal/mol with respect to critical point **o**.

**Table 6:** Total energies (E) ^a^, zero-point energies (ZPE) ^a^, imaginary frequencies (ν) ^b^, spin contamination (S^2^), and relative energies (ΔE) ^c^ of critical points of the triplet-state PES of the thermolysis reaction of 3,3,6-trimethyl-1,2,4,5-tetroxane (TMT) in position C-6 axial (ax) and equatorial (eq) isomers. Acetaldehyde as the first intermediate product (Figure 5a) and acetone as the second product (Figure 6a).

**Structure**	E ^a^		ZPE ^a^		ν ^b^		S^2^		ΔE ^c^	
	C-6ax	C-6eq	C-6ax	C-6eq	C-6ax	C-6eq	C-6ax	C-6eq	C-6ax	C-6eq
Step 1										
**o**	−497.100010	497.101706	0.154987	0.154850			2.009	2.000	0.00	0.00
TS**_ob′_**	−497.070759	−497.071879	0.152803	0.152552	888	875	2.000	2.000	16.98 ^d^	15.13 ^d^
**b′**	−497.115218	−497.115340	0.151676	0.151731			2.021	2.000	−11.62 ^e^	−10.51 ^e^
Step 2										
**b**	−343.320980	−343.320980	0.093508	0.093508			2.000	2.000	0.00	0.00
TS**_bp_**	−343.310328	−343.310328	0.090696	0.090696	4612	4612	2.000	2.000	6.88	6.88
**p**	−343.389853	−343.389853	0.090578	0.090578			2.000	2.000	−43.10	−43.10

^a^ Hartrees. ^b^ Imaginary frequencies in cm^−1^. ^c^ Relative energies in kcal/mol, $\Delta E=[E_{total}+ZPE](o/b)-[E_{total}+ZPE](i)$, where ***i*** represents any critical point of the species in the table. ^d^ The relative energies with respect to Sc are 28.78 and 31.03 kcal/mol for the axial and equatorial isomers, respectively. ^e^ The relative energies with respect to Sc are −3.67 and −4.33 kcal/mol for the axial and equatorial isomers, respectively.

**Table 7:** Total energies (E) ^a^, zero-point energies (ZPE) ^a^, imaginary frequencies (ν) ^b^, spin contamination (S^2^), and relative energies (ΔE) ^c^ of critical points of the triplet-state PES of the thermolysis reaction of 3,3,6-trimethyl-1,2,4,5-tetroxane (TMT) in position C-6 axial (ax) and equatorial (eq) isomers. Acetone as the first intermediate product (Figure 5b) and acetaldehyde as the second product (Figure 6b).

**Structure**	E ^a^		ZPE ^a^		ν ^b^		S^2^		ΔE ^c^	
	C-6ax	C-6eq	C-6ax	C-6eq	C-6ax	C-6eq	C-6ax	C-6eq	C-6ax	C-6eq
Step 1										
**o**	−497.100010	−497.101706	0.154987	0.154850			2.009	2.000	0.00	0.00
TS**_ob′_**	−497.073526	−497.075179	0.152727	0.152551	837	848	2.000	2.000	15.20 ^d^	15.20 ^d^
**b′**	−497.121021	−497.116653	0.151932	0.151264			2.021	2.000	−15.10 ^e^	−11.63 ^e^
Step 2										
**b**	−304.018708	−304.019876	0.065059	0.064864			2.000	2.000	0.00	0.00
TS**_bp_**	−304.003558	−303.004328	0.062416	0.061914	3784	3503	2.000	2.000	7.90	7.84
**p**	−304.080579	−304.080668	0.061737	0.061720			2.000	2.000	−40.91	−40.12

^a^ Hartrees. ^b^ Imaginary frequencies in cm^−1^. ^c^ Relative energies in kcal/mol, $\Delta E=[E_{total}+ZPE](o/b)-[E_{total}+ZPE](i)$ where ***i***, represents any critical point of the species in the table. ^d^ The relative energies with respect to Sc are 27.16 and 28.95 kcal/mol for the axial and equatorial isomers, respectively. ^e^ The relative energies with respect to Sc are −2.16 and −2.12 kcal/mol for the axial and equatorial isomers, respectively.

**Table 8:** Total energies (E) ^a^, zero-point energies (ZPE) ^a^, imaginary frequencies (ν) ^b^, spin contamination (S^2^), and relative energies (ΔE) ^c^ of the critical points of the triplet PES for the thermolysis reaction of 3,3,6,6-tetramethyl-1,2,4,5-tetroxane (ACDP).

**Structure**	E ^a^	ZPE ^a^	Ν ^b^	S^2^	ΔE ^c^
**qc**	−536.401787	0.183464		2.000	1.39 ^d^
Step 1					
**o**	−536.403895	0.183362		2.000	0.00
TS**_ob′_**	−536.377483	0.180690	844	2.000	15.13 ^e^
**b′**	−536.425114	0.180509		2.000	−15.12 ^f^
Step 2					
**b**	−343.320980	0.093508		2.000	0.00
TS**_bp_**	−343.219631	0.090696	4612	2.000	6.88
**p**	−343.389843	0.090578		2.000	−43.10

^a^ Hartrees. ^b^ Imaginary frequencies in cm^−1^. ^c^ Relative energies in kcal/mol, $\Delta E=[E_{total}+ZPE](o/b)-[E_{total}+ZPE](i)$, where ***i*** represents any critical point of the species in the table. ^d^ With respect to T-**o**. ^e^ The relative energy with respect to Sc is 28.70 kcal/mol. ^f^ The relative energy with respect to Sc is −1.56 kcal/mol.

**Table 9:** Spectroscopic characterization of the reactant. *E*_VA_ corresponds to the CASPT2 (12-in-8)/ANO-L-VDZP vertical absorption energy (eV) of FDP. Main nature of the transitions and the corresponding weight of the electronic configurations are also shown (see orbitals in Figure 7).

State	Nature	Weight (%)	E_VA_ (eV)
S_1_	n_3_→*σ*_1_*	78	5.09
S_2_	n_2_→*σ*_2_*	74	5.14
S_3_	n_4_→*σ*_1_*	80	5.78
T_1_	n_3_→*σ*_1_*	39	3.87
	n_2_→*σ*_2_*	26	
T_2_	n_2_→*σ*_1_*	34	3.93
	n_3_→*σ*_2_*	30	
T_3_	n_4_→*σ*_1_*	54	4.42
	n_1_→*σ*_2_*	27	
T_4_	n_4_→*σ*_2_*	45	4.81
	n_1_→*σ*_1_*	35	

**Table 10:** SOC complex vector norms (see Computational Details) in cm^−1^ for the tetraoxane series. Data obtained with the CASSCF(12,8)/ANO-L-VDZP method. Note that T_i_ encompasses the three *m_s_* components of the triplet state.

	FDP	MFDP (ax)	MFDP (eq)	DMT (aa)	DMT (ae)	DMT (ee)	TMT (aae)	TMT (aee)	ACDP
T_1_-S_0_	0.03	0.06	0.05	0.10	0.09	0.07	0.15	0.13	0.21
T_2_-S_0_	21.12	65.27	66.46	23.72	41.81	27.77	61.30	57.93	33.84
T_3_-S_0_	96.04	76.50	72.42	95.29	88.91	94.24	76.58	79.27	91.94
T_4_-S_0_	0.23	0.13	0.20	0.12	0.14	0.16	0.41	0.23	0.65
T_1_-S_1_	21.13	62.65	64.16	23.67	32.08	27.76	51.95	46.56	33.71
T_2_-S_1_	0.10	0.50	0.43	0.19	0.41	0.16	1.01	0.75	0.33
T_3_-S_1_	0.44	0.04	0.51	1.85	0.84	0.17	1.95	1.10	3.13
T_4_-S_1_	21.28	62.25	63.81	23.97	32.15	26.74	51.69	46.49	34.10
T_1_-S_2_	96.12	75.90	74.57	95.51	93.00	94.34	83.46	86.61	92.26
T_2_-S_2_	1.22	0.21	0.66	0.37	0.18	0.83	1.25	0.34	1.16
T_3_-S_2_	0.03	0.48	0.43	0.03	0.26	0.03	0.48	0.44	0.00
T_4_-S_2_	95.39	75.08	73.88	94.35	91.97	93.39	82.53	85.61	91.23
T_1_-S_3_	0.14	0.10	0.12	0.35	0.22	0.03	0.60	0.35	0.93
T_2_-S_3_	21.26	64.98	66.18	23.90	41.66	27.99	61.60	57.71	33.99
T_3_-S_3_	95.47	72.87	71.87	94.53	88.19	93.49	76.08	78.64	91.48
T_4_-S_3_	0.17	0.21	0.20	0.29	0.26	0.24	0.36	0.32	0.48

## Data Availability

The original contributions presented in the study are included in the article, and further inquiries can be directed to the corresponding authors.
